# Comment on “The State of Exosomes Research: A Global Visualized Analysis” and “Current Research Trends in Traditional Chinese Medicine Formula: A Bibliometric Review from 2000 to 2016”

**DOI:** 10.1155/2019/6217925

**Published:** 2019-12-05

**Authors:** Yuh-Shan Ho

**Affiliations:** Trend Research Centre, Asia University, No. 500, Lioufeng Road, Wufeng, Taichung 41354, Taiwan

“The State of Exosomes Research: A Global Visualized Analysis” [1] and “Current Research Trends in Traditional Chinese Medicine Formula: A Bibliometric Review from 2000 to 2016” [2] were recently published in *BioMed Research International* and *Evidence-Based Complementary and Alternative Medicine*, respectively.

Wang et al. stated in Section 2.1 that “Publication information from the Web of Science (SCI-Expanded)” and in Section 2.2 that “the research terms” were as follows: theme = exosome^*∗*^ AND publishing year = (from 1994 to 2017) AND Language = (only English) AND Document types = (REVIEW OR ARTICLE)” [1].

Using the methods applied by Wang et al. [1], we found 7,323 publications which are very different from 4,960 in the original paper [1]. To improve the author's method, appropriate keywords should be applied. A total of 7,323 English publications including 5,682 articles and 1,641 reviews were found in SCI-EXPANDED (updated on April 26, 2019) from 1994 to 2017 with keywords such as “exosomes” and “exosome” in the topic including document title, abstract, author keywords, and *KeyWords Plus* were taken into consideration.

Chen et al. stated in 2.1. Search Strategy that “Papers published from 2000 to 2016 were tracked by the keywords “Traditional Chinese Medicine”, “Traditional Chinese Medicine Formula”, and “Chinese Herb Formula” for inclusion in analysis and summarization, based on their presence in the Web of Science database [2].

Web of Science from 2000 to 2016 were applied as mentioned in the original paper [2]. In total, 42,270 and 20,909 articles were found by using searching keywords (traditional Chinese medicine or traditional Chinese medicine formula or Chinese herb formula) and (“traditional Chinese medicine” or “traditional Chinese medicine formula” or “Chinese herb formula”), respectively. These results show a huge difference (57% of 26,917 articles) from the results in the original paper “A total of 26,917 TCMF-related articles were published in the period from 2000 to 2016” [2]. Furthermore, Chen et al. also stated in Section 2.1 that “it contains more than 12,000 core academic journals with the most influential research fields in natural sciences, engineering technology, and biomedicine” [2]. This information cannot be found in the cited reference [3] in the original paper [2].

Web of Science databases (https://clarivate.com/products/web-of-science/databases/) includes

1. Web of Science Core Collection
2. Specialist Collection
    BIOSIS Citation Index
    BIOSIS Previews
    Biological Abstracts
    Zoological Record
    MEDLINE
    CAB Abstracts, CABI Global Health
    Inspec
    FSTA
3. Regional Collection
    Chinese Science Citation Database
    Russian Science Citation Index
    KCI Korean Journal Database
    SciELO Citation Index
4. Data Collection
    Data Citation Index
5. Patent Collection
    Derwent Innovations Index (DII)

Web of Science Core Collection: Citation Indexes includes

1. Science Citation Index Expanded (SCI-EXPANDED)
2. Social Sciences Citation Index (SSCI)
3. Arts and Humanities Citation Index (A&HCI)
4. Conference Proceedings Citation Index-Science (CPCI-S)
5. Conference Proceedings Citation Index-Social Science and Humanities (CPCI-SSH)
6. Book Citation Index-Science (BKCI-S)
7. Book Citation Index-Social Sciences and Humanities (BKCI-SSH)
8. Emerging Sources Citation Index (ESCI)

Web of Science Core Collection: Chemical Indexes

1. Current Chemical Reactions (CCR-EXPANDED)
2. Index Chemicus (IC)

Web of Science Core Collection includes multidisciplinary information from over 18,000 high-impact journals, over 180,000 conference proceedings, and over 80,000 books from around the world (https://clarivate.com/products/web-of-science/databases/).

Authors should choose appropriate databases based off of their topic instead of choosing random databases for their bibliometric research. For instance, Emerging Sources Citation Index (ESCI) complements the highly selective indexes by providing earlier visibility for sources under evaluation as part of SCIE, SSCI, and A&HCI's rigorous journal selection process (http://liu.brooklyn.libguides.com/az.php?a=e). Web of Science Core Collection: Chemical Indexes as well as SSCI, A&HCI, ESCI, CPCI-S, CPCI-SSH, BKCI-S, and BKCI-SSH are inappropriate for “Current Research Trends in Traditional Chinese Medicine Formula: A Bibliometric Review from 2000 to 2016” [2].

The SCI-EXPANDED is designed mainly for researchers to find published literature studies, not for bibliometric studies [4–6]. Thus, it is always necessary to use an accurate bibliometric treatment when using the Web of Science database [4–6]. Fu and Ho [7] pointed out that the documents, which can only be searched out by *KeyWords Plus*, were irrelevant to “exosomes” in Wang et al.'s paper [1] and “traditional Chinese medicine formula” in Chen et al.'s paper [2]. Due to biases by using SCI-EXPANDED for bibliometric studies, Ho's group was the first to propose “front page” (including the article title, the abstract, and the author keywords) as a filter to improve the bibliometric method [8–10].

In the case of Wang et al.'s paper [1], 1,371 publications (19% of the 7,323 publications) did not contain searching keywords (“exosomes” and “exosome”) in their “front page”. For instance, classic article [11] with 1,000 or more total citations from Web of Science Core Collection since publication to the end of the most recent year, “The MicroRNA spectrum in 12 body fluids” [12]; and highly cited articles [13] with 100 or more total citations “Initial genome sequencing and analysis of multiple myeloma” [14] and “Secreted monocytic miR-150 enhances targeted endothelial cell migration” [15].

In the case of Chen et al.'s paper [2], after our pre-study, one appropriate method is to use SCI-EXPANDED with searching keywords (“Chinese medicine formula” or “Chinese medicine formulas” or “Chinese medicine formulation” or “Chinese medicine formulations” or “Chinese medicine formulae” or “Chinese herb formula” or “Chinese herb formulas” or “Chinese Herb Formulation”) from 2000 to 2016. Document type of “article” was considered. This method resulted in 315 articles. Altogether, 310 articles (98% of the 315 articles) had searching keywords in their “front page” and five articles (1.6% of the 315 articles) had no any searching keywords in their “front page”.

Furthermore, typical evidence was found in Table 7 [1]. The top 20 highly cited Traditional Chinese Medicine Formula-related articles were found in Chen et al.'s original paper [2]. Some articles did not include “formula” in their “front page” in Table 7 in the original paper [1]; for example, highly cited article entitled “Multiple chromatographic fingerprinting and its application to the quality control of herbal medicines” [16]; “Comparative pharmacokinetics of baicalin after oral administration of pure baicalin, *Radix scutellariae* extract and Huang-Lian-Jie-Du-Tang to rats” [17]; and “An approach to develop two-dimensional fingerprint for the quality control of *Qingkailing* injection by high-performance liquid chromatography with diode array detection” [18].

Published articles by for example Li and Nan [19], Liu and Liao [20], Liang and Liu [21], Li et al. [22], and Gao and Ruan [23] with the same problem can also be found in recent years. In order to stop the proliferation of the problem, comments have been made in *Environmental Science and Pollution Research* [4], *Renewable and Sustainable Energy Reviews* [24], *Journal of Soils and Sediments* [6], and *Journal of Foot and Ankle Surgery* [25]. However, the same problem is constantly being presented by authors and published by journals such as *International Journal of Environmental Research and Public Health* [26, 27], *Sustainability* [28, 29], *Journal of Cleaner Production* [30, 31], and *Environmental Science and Pollution Research* [32, 33]. Editors might reject similar comments like this. However it is more important to stop publishing papers with the same problem, at the same time, letting authors and readers know the serious problem behind these published papers.

Wang et al. [1] and Chen et al. [2] used inappropriate methods to publish bibliometric papers in *BioMed Research International* and *Evidence-Based Complementary and Alternative Medicine*. This may result in misleading readers of the journals. From my prospective, authors should have used an appropriate bibliometric method and filter for their studies, thereby providing greater accuracy results for their bibliometric studies.
